# Abderrazak Hajjioui: shining a light on rehabilitation

**DOI:** 10.2471/BLT.22.031122

**Published:** 2022-11-01

## Abstract

Abderrazak Hajjioui talks to Tatum Anderson about the need to prioritize multidisciplinary rehabilitation education, practice and research.

*Q: When did rehabilitation become the prime focus of your career?*

A: During my medical school training and subsequent internships. I noticed that Moroccan practitioners tended to be more interested in patients’ diseases than in the patients themselves or in the quality of life they were going to have once they had been “treated” or “cured”. It really struck me when I was working in the neurology department of the Ibn Sina University hospital in Rabat; we would receive stroke patients, make a diagnosis, give them medication and then send them back home. This despite many of the patients being barely able to move or walk or take care of themselves. Witnessing those outcomes, and the complaints of the patients and their families, steered me towards a greater interest in rehabilitation. 

*Q: You were one of the first Moroccan doctors to specialize in rehabilitation. What kind of challenges did you face?*

A: The most obvious was the lack of specialists in the domain to teach me and my junior colleagues. I spent my first year of residency in Morocco and then spent several years in France, where I was able to pursue my specialization at the University of Versailles Saint-Quentin and René Descartes University in Paris. Once qualified, there was an opportunity for me to lead the hospital’s rehabilitation programme in Fez, a responsibility I was happy to take on.

*Q: Have others in Morocco followed in your path?*

A: Not as many as I would have expected. Unfortunately, rehabilitation is not a priority in Morocco. This is expressed in many ways, but notably in education. For example, until recently we didn’t have a course or programme on rehabilitation in the national undergraduate medical curriculum. Lacking exposure to rehabilitation, very few graduating doctors opt for it as a career choice in their residencies.

*Q: What are the main obstacles to developing the field of rehabilitation in Morocco?*

A: As in many low- and middle-income countries, lack of resources is a factor, but so is the distribution of those resources – the decisions taken about where resources should go. We are willing to spend money on emergency surgery, and acute medicine, but not on rehabilitation. We send patients home from acute medicine or emergency departments without rehabilitation, and they suffer as a result, often dying from complications.

“The priority is always given to acute care.”

*Q: Can you say more about that?*

A: Research my team and I did on the outcomes and quality of life of people with spinal cord injury and with brain injury – the most common acute conditions in Morocco – revealed that roughly half of the patients die of complications in the first year after the acute phase, and those are the patients who have received some limited rehabilitation support. It is just not acceptable that we send stroke patients home after a short hospital stay with a prescription for physiotherapy at city-based ambulatory rehabilitation centres that generally lack capacity and rehabilitation infrastructure. Sometimes they get a wheelchair, sometimes they don’t. And the situation is not going to get any better as the demand for rehabilitation continues to increase.

*Q: What is driving that increase in Morocco?*

A: As in many countries, the ongoing epidemiological transition towards increased prevalence of noncommunicable diseases, driven in part by increasing obesity and sedentary lifestyles, is driving increased demand. So too is population ageing, driven by increased life expectancy – and increased years lived with disability – and declining fertility. According to the WHO/IHME Rehabilitation Needs Estimator (a web-based tool developed by the World Health Organization with the Institute for Health Metrics and Evaluation (IHME) that provides global-, regional- and country-level data visualizations of the estimated need for rehabilitation globally) in 2019, 11 million people out of a population of 37 million in Morocco experienced conditions that could benefit from rehabilitation.

*Q: What can be done to meet the demand for rehabilitation services?*

A: First, we need to change the vision and perceptions of decision-makers with regard to rehabilitation. Working in collaboration with the Ministry of Health, my colleagues and I are advocating to improve the awareness of rehabilitation, as part of universal health coverage, highlighting rehabilitation’s importance to health and well-being. Of course, the medical profession perceptions of rehabilitation must also change. I am talking about my fellow professionals. For example, at my hospital we have funds allocated for capacity development and could develop rehabilitation departments. But the priority is always given to acute care. So, we need to do more to raise awareness among doctors and decision-makers.

*Q: You are a clinician and an educator. What, if anything, needs to change in the way rehabilitation is taught in universities?*

A: First it needs to be made a core part of educational curricula. I have worked with a network of medical schools to develop a module of 10 hours on rehabilitation and disabilities. The module was introduced into the national curriculum in 2015. I am also now working with the National School of Public Health to incorporate a module on rehabilitation into a course on health facilities management for future policy-makers. In addition, I launched two university diplomas: one concerning cardiovascular and pulmonary rehabilitation, chronic disease and ageing, and the other concerning the neuro-rehabilitation of adults. Rehabilitation education should also encourage multidisciplinary approaches. As part of our university diplomas, we invite rehabilitation teams comprised of doctors, physiotherapists, speech therapists, occupational therapists, orthotists and prosthetists and nurses to take the same course on how to manage, for example, stroke survivors from the acute phase to their reintegration into the community. This collaboration is essential to develop shared language and approaches and to build our teams. It’s important to remember that education is not limited to university or medical school. One of the biggest challenges we face is how to provide training for the different kinds of rehabilitation professionals once they graduate.

*Q: Is that because of a lack of specialists or a lack of rehabilitation centres in which to train?*

A: Both. For example, there are no post-acute inpatient rehabilitation centres in the public health system. Rehabilitation should begin as early as possible after surgery and is best delivered through a versatile, multidisciplinary inpatient and outpatient approach. Unfortunately, in Morocco, as in many lower-income countries, rehabilitation services are only delivered as ambulatory and thus they are not accessible to the patients who are most disabled. In the public health system, there are about 24 000 beds for acute care, with an occupancy rate of less than 65%, but zero beds for rehabilitation patients. The same is true of more specialized rehabilitation capacity. I know many physiotherapists who want to get specialized training in neurological rehabilitation, for example, but we don’t have any neurological rehabilitation departments to send them to. This lack of capacity and lack of opportunities discourages graduates from specializing in rehabilitation and is one of the reasons why so few medical graduates are recruited to train and work in rehabilitation.

“We send stroke patients home after a short hospital stay.”

*Q: To what extent is the brain drain a problem?*

A: It is a big problem here, especially with France being so close and so many of our graduates speaking French as a second language. As I mentioned, I pursued my studies in France and could have stayed there. I chose to come back. Many do not. So, yes, the migration of young graduates and the best rehabilitation professionals to the most developed countries is a big problem, as is the unequal distribution between the different cities and regions of the Kingdom of Morocco.

*Q: How important is developing local research capacity to strengthen rehabilitation?*

A: Research is of crucial importance and an area of great concern. We need researchers to publish data about rehabilitation and health issues in the Moroccan context to establish the evidence base needed to guide public health policy. We also need rehabilitation research that answers the questions being raised by health policy-makers regarding the needs of the population. Unfortunately, despite efforts made in recent years, research in rehabilitation in Morocco is still limited by several barriers. These include the absence of a research community, lack of funding, insufficient experience of researchers, and barriers to access such as high publication fees. We also have a problem of quality which is in part a reflection of conditions in the field. How can we produce a good paper about stroke rehabilitation when we don’t have a stroke rehabilitation department? It is a chicken and egg situation: research drives capacity development which drives better research. Of course, this problem is not limited to the Moroccan health system. We need to train, strengthen and encourage researchers working across low- and middle-income countries worldwide. It’s worth remembering that an estimated 80% of people with disabilities live in these countries. It is for this reason that I am also committed to regional capacity development and am working with WHO and many nongovernmental organizations to advance that agenda.

*Q: What are your hopes for the future?*

A: I would like to see rehabilitation services, both specialized and general, established at different levels of the health system in Morocco. I would also like to see rehabilitation embraced as a core component of SDG 3 (the SDG health goal). Rehabilitation not only reduces the burden of disability – it enables people living with disability to fully participate in the life and work of their communities and society as a whole. As such, it is clearly vital to the realization of global health and well-being and beyond that the overarching goal of sustainable development.

